# *In Situ* Detection of Antibiotic Amphotericin B Produced in *Streptomyces nodosus* Using Raman Microspectroscopy

**DOI:** 10.3390/md12052827

**Published:** 2014-05-13

**Authors:** Rimi Miyaoka, Masahito Hosokawa, Masahiro Ando, Tetsushi Mori, Hiro-o Hamaguchi, Haruko Takeyama

**Affiliations:** 1 Department of Life Science and Medical Bioscience, Waseda University, 2-2 Wakamatsu-cho, Shinjuku-ku, Tokyo 162-8480, Japan; E-Mails: r-miyaoka@ruri.waseda.jp (R.M.); m.hosokawa@aoni.waseda.jp (M.H.); moritets@aoni.waseda.jp (T.M.); 2 Consolidated Research Institute for Advanced Science and Medical Care, Waseda University, 513, Wasedatsurumaki-cho, Shinjuku-ku, Tokyo 162-0041, Japan; E-Mails: mando@aoni.waseda.jp (M.A.); hhama@nctu.edu.tw (H.H.); 3 Core Research for Evolutionary Science and Technology (CREST), Japan Science and Technology Agency (JST), 5, Sanbancho, Chiyoda-ku, Tokyo 102-0075, Japan; 4 Institute of Molecular Science and Department of Applied Chemistry, National Chiao Tung University, 1001 To Hsuch Road, Hsinchu 300, Taiwan

**Keywords:** Raman microspectroscopy, *in situ* detection, antibiotics, secondary metabolites, actinomycetes

## Abstract

The study of spatial distribution of secondary metabolites within microbial cells facilitates the screening of candidate strains from marine environments for functional metabolites and allows for the subsequent assessment of the production of metabolites, such as antibiotics. This paper demonstrates the first application of Raman microspectroscopy for *in situ* detection of the antifungal antibiotic amphotericin B (AmB) produced by actinomycetes—*Streptomyces nodosus*. Raman spectra measured from hyphae of *S. nodosus* show the specific Raman bands, caused by resonance enhancement, corresponding to the polyene chain of AmB. In addition, Raman microspectroscopy enabled us to monitor the time-dependent change of AmB production corresponding to the growth of mycelia. The Raman images of *S. nodosus* reveal the heterogeneous distribution of AmB within the mycelia and individual hyphae. Moreover, the molecular association state of AmB in the mycelia was directly identified by observed Raman spectral shifts. These findings suggest that Raman microspectroscopy could be used for *in situ* monitoring of antibiotic production directly in marine microorganisms with a method that is non-destructive and does not require labeling.

## 1. Introduction

Various secondary metabolites, which have broad functions—antibacterial, antifungal, antiviral, antitumor, and antiprotozoal—have been isolated from different microbes found in terrestrial soils and marine sediments [1,2,3]. One third of the 22,500 known microbial metabolites are the secondary metabolites of actinomycetes, particularly *Streptomyces* species [4]. *Streptomyces* fermentation products are rich sources of antibiotics, such as antibacterial streptomycin (*Streptomyces griseus*) [5], kanamycin (*Streptomyces kanamyceticus*) [6], tetracycline (*Streptomyces rimosus*) [7], antifungal amphotericin B (*Streptomyces nodosus*) [8], antitumor actinomycin (*Streptomyces antibioticus*) [9], and doxorubicin (*Streptomyces peucetius)* [10].

Currently, a large variety of antibiotics are produced in microbial fermentation processes or derived by chemical modification of microbial products. Because of continued demand for their cost-effective production, rapid and efficient assessments of the fermentation product are required for the screening of microbial culture conditions. In general, the metabolite contents within microbial cells are invasively analyzed by solvent extraction-based methods. The purified microbial extracts are subsequently analyzed by GC-MS or NMR to determine their chemical formula and abundance. These processes are invasive, time-consuming, laborious, and require a substantial amount of microbe cultures. Moreover, the conventional methods cannot provide real-time information for assessment and improvement of the fermentation parameters. 

Meanwhile, chemical screening of microbial metabolites is a starting point for discovery of new drug candidates from environmental microbes. For example, marine sponges are known to harbor a massive consortium of uncultivated bacteria, which produce medically important natural products [11,12]. To explore the metabolic potential of these microbes, we have applied metagenomic and single-cell-based approaches to identify target metabolite producers [13,14]. However, because of the lack of appropriate probes that enable *in situ* identification of microbial metabolites, identification of new drug candidates is dependent on the analysis of whole sponge extracts by a solvent extraction-based method. Therefore, in conjunction with the demand for novel drugs from environmental sources and cost-effective production, a non-destructive technique for compositional analysis of microbial secondary metabolites is required.

Raman spectroscopy provides characteristic information on the molecular structure of metabolites and does not require any sample pretreatment such as dye labeling or genetic manipulation, thus allowing for rapid and low-invasive observations. Raman spectroscopy can be used to investigate biological samples—plants [15], animals [16] or human tissues [17]. Indeed, Raman spectroscopy has been utilized to quantify the level of penicillin from fermentation broths for in-line analysis [18]. Moreover, in combination with optical microscopy, Raman microspectroscopy provides high space-resolved information of human cells [19], fungi [20], or bacteria, including *Streptomyces* species [21]. Our research group has carried out time- and space-resolved Raman imaging of living yeast cells using confocal Raman microspectroscopy [22,23,24]. The Raman images of cells show that the distribution of lipids and proteins vary during cell division cycles. Because of these capabilities, recent reports reveal the distribution of secondary metabolites—pigment in plants [25] and green macroalgae [26]—using FT-Raman microspectroscopy. Since secondary metabolites such as antibiotics generally have diverse and distinctive chemical structures, we postulated that Raman imaging has the potential to distinguish antibiotics from other biomolecules in living cells without labeling or extraction.

Here, we report the first demonstration of Raman imaging for *in situ* detection of microbially derived antibiotics in living microbial cells. In this study, *S. nodosus*—known to produce Amphotericin B (AmB)—was analyzed as a model for antibiotic-producing actinomycetes. AmB belongs to polyene antibiotics, has a broad spectrum of activity, and has shown efficacy against candidiasis, cryptococcosis, aspergillosis, histoplasmosis, blastomycosis, coccidioidomycosis, zygomycosis, sporotrichosis, fusariosis, and phaeohyphomycosis [27]. The Raman spectra of AmB has a strong band, due to the phenomenon of resonance enhancement, at 1559 cm^−1^ that corresponds to the C=C symmetric vibration of the polyene chain [28]. The Raman intensities of this specific band correlate with the antifungal activity and the abundance of antibiotic. To evaluate antibiotic production, we applied Raman imaging of AmB to live *S. nodosus* cells. Here, we demonstrate the ability to detect and image *in situ* distribution of antibiotics within mycelia and individual hyphae with high spatial resolution using Raman microspectroscopy. This study demonstrates the capability of Raman imaging for non-destructive screening of antibiotic producers and the potential of Raman microspectroscopy, as an in-line monitoring technique, to be a tool for use in antibiotic production from industrial scale fermentation cultures.

## 2. Results and Discussion

### 2.1. Raman Spectra of AmB Produced in S. nodosus

In this study, *in situ* detection of AmB produced within the actinomycetes *S. nodosus* was conducted using a laboratory-built confocal Raman microspectrometer. To analyze Raman spectra at single-cell resolution, the Raman microspectrometer was equipped with a 532-nm laser, an inverted microscope with a 100 × 1.4 NA lens, a spectrometer, and a charge-coupled device (CCD) detector [24]. The lateral and depth spatial resolutions of the Raman imaging system were 0.3 and 2.6 μm, respectively. 

**Figure 1:**
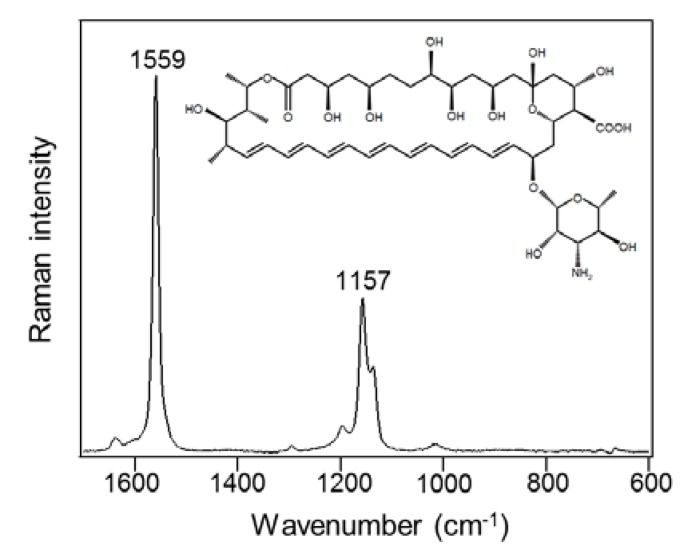
Raman spectrum of standard amphotericin B in dimethyl sulfoxide (30 mg/mL).

AmB is an elongated, cyclic molecule consisting of hydrophilic polyhydroxyl and hydrophobic polyene domains (Figure 1 inset). The hydrophobic polyene domain promotes binding to and insertion into fungal, lipid bilayer membranes. As shown in Figure 1, the Raman spectrum of standard AmB (30 mg/mL) shows the specific bands due to the hydrophobic polyene domain at 1559 cm^−1^, corresponding to the C=C stretch, and 1157 cm^−1^, corresponding to the C-C stretch due to resonance Raman scattering [29].

**Figure 2:**
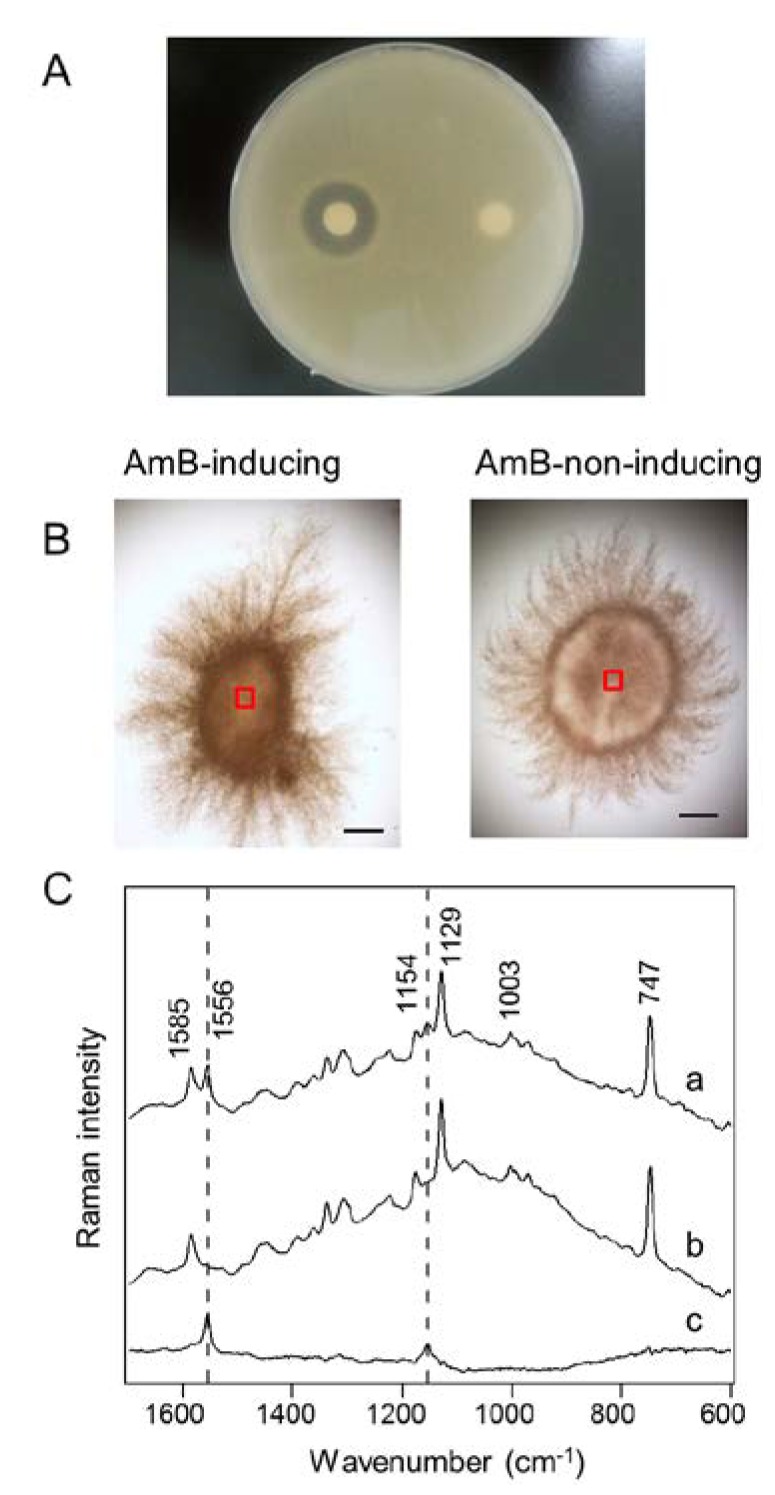
Raman microspectroscopic analysis of *S. nodosus* mycelia. (**A**) Evaluation of antifungal activity by the paper disc assay. The paper discs soaked with the extracts of *S. nodosus* mycelia cultivated under amphotericin B (AmB)-inducing (the left disk) and -non-inducing (the right disk) conditions were placed on an agar plate with *Candida albicans*; (**B**) Bright field images of *S. nodosus* mycelia cultivated in AmB-inducing medium and AmB-non-inducing medium. The Raman spectra were acquired from the areas indicated in the red boxes (30 μm × 30 μm). Scale bar = 200 μm; (**C**) Raman spectra were obtained from the centers of mycelia. Difference spectrum (**c**) between inducing (**a**) and non-inducing (**b**) conditions was obtained. Dashed lines indicate the AmB-specific bands at 1154 and 1556 cm^−1^.

After cultivation of *S. nodosus* under AmB production-inducing or -non-inducing conditions, the mycelia derived from single spores were recovered from the culture media. The synthesis of AmB was also confirmed by a paper disc assay (Figure 2A). Antifungal activity against *Candida albicans* was successfully confirmed in the cell extract obtained from AmB-inducing medium, while no activity was observed from AmB-non-inducing medium. In the Raman microspectroscopic analysis, the same cell samples were analyzed without cell destruction. As shown in Figure 2B, the mycelia were approximately 1.5 mm in size after cultivation. The centers of the mycelia (900 μm^2^; indicated by red boxes in Figure 2B) were selected for Raman microspectroscopic analysis. Since the depth resolution of confocal Raman microspectroscopy is smaller than the mycelia, the Raman intensity depends on constituent molecules within focal points. In the Raman spectra of *S. nodosus* (Figure 2C), three notable bands (747, 1129, and 1585 cm^−1^) of cytochrome b and c can be found, as described previously in resonance Raman spectra [30]. In addition, protein can be found at 1003 cm^−1^, corresponding to phenylalanine ring breathing. Cytochrome and protein are commonly analyzed by Raman microspectroscopy to identify cell shapes and to evaluate respiration bioactivity [24,30]. Based on the Raman spectra obtained from the cells cultivated under AmB-inducing conditions (Figure 2C (a)), we determined that two bands at 1556 and 1154 cm^−1^ could be assigned to AmB. To confirm the assignments, we further compared the Raman spectra obtained from the AmB-inducing and AmB-non-inducing conditions (Figure 2C (b)). The difference spectrum (Figure 2C (c)) suggests that these bands—1556 and 1154 cm^−1^—could be observed only in the cells cultivated under AmB-inducing conditions, even though the peaks shifted toward lower frequencies as compared with the spectrum of standard AmB. Moreover, no obvious changes of other cellular components were observed between AmB production-inducing and -non-inducing conditions. On the basis of these results, we assigned these two bands at 1556 and 1154 cm^−1^ to AmB within live cells for the subsequent experiments. 

### 2.2. Prediction of the Molecular State of AmB from Raman Peak Shift

Since polyene antibiotics are poorly soluble in aqueous solvents with water solubility of <1 mg/L at physiological pH (pH 6–7) [31], AmB self-associates and aggregates in water, owing to its amphipathic nature [32]. The molecular aggregation of AmB, which is induced by various physical and chemical factors—concentration, solvent, temperature, and pH—has influence on the spectral shift [33]. The Raman band corresponding to the C=C stretch shows sensitivity to structural changes, resulting in an intensity decrease and a slight shift of the predominant band [28]. Gagos *et al.* reported that aggregated AmB at pH 7 exhibits a Raman peak shift to lower frequencies by 2 cm^−1^, compared to that of monomeric AmB at pH 12 [28]. In accordance with these findings, the specific bands of AmB at 1556 cm^−1^ obtained from the mycelia (Figure 2) have a spectral shift of 3 cm^−1^ toward lower frequencies, as compared to the standard AmB band, which was solubilized in DMSO in a dimeric state (Figure 1). Therefore, we speculate that AmB accumulates in the molecular aggregate state, which results in the peak shifts visible on the Raman spectra.

**Figure 3:**
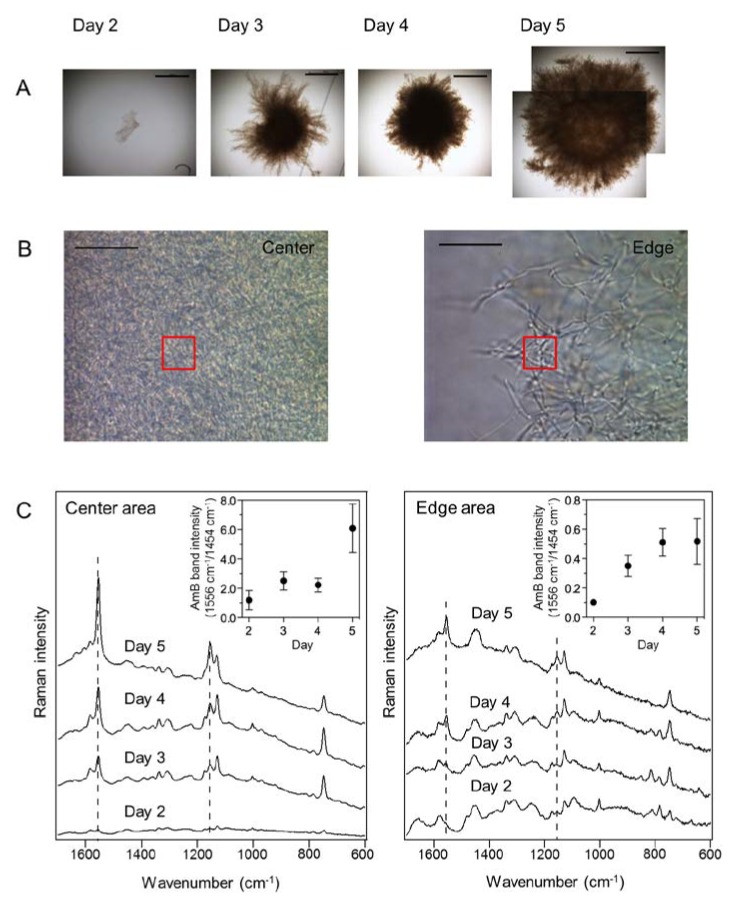
*In situ* time-course analysis of amphotericin B (AmB) production in *S. nodosus* mycelia. (**A**) Bright field images of *S. nodosus* mycelia cultivated for 5 days. Scale bar = 500 μm; (**B**) Magnified images of the center and edge areas of the mycelia at day 5 used for Raman spectroscopy measurements. The Raman spectra were acquired from the areas indicated in the red boxes (10 μm × 10 μm). Scale bar = 20 μm; (**C**) Time-dependent changes of averaged Raman spectra obtained from the center and edge areas of mycelia. Ten mycelia were analyzed at each time point. Dashed lines indicate the AmB-specific bands at 1154 and 1556 cm^−1^. Insets show the changes of AmB-specific band intensities, which are normalized by the Raman band that corresponds to biomass (1454 cm^−1^).

### 2.3. In Situ Time-Course Analysis of AmB Production in S. nodosus Using Raman Microspectroscopy

To monitor time-dependent changes in AmB production, the Raman spectra of center and edge areas of mycelia were measured on days 2–5 (Figure 3A). In each time point, 10 mycelia were sampled from culture medium and then analyzed by Raman microspectroscopy. The averaged Raman spectra were obtained from the fixed volume of 10 μm × 10 μm × 2.6 μm for the *x*, *y*, and *z* axes at each time point, during a period of mycelia growth (Figure 3B). Hence, the changes of the Raman intensities correlate with the fluctuation of constituent molecular numbers within fixed focal planes. The averaged spectrum obtained from the center area of 10 mycelia shows the AmB-specific Raman bands from day three, and the band intensity of AmB increased as a function of cultivation time (Figure 3C). As compared to the averaged Raman spectra of the edge areas, those of the center areas show significant changes of Raman intensity corresponding to AmB. These results indicate that AmB accumulates predominantly in the center of the mycelium, which is dense with cells, as a function of cultivation time. Therefore, Raman microspectroscopy could be employed for non-destructive monitoring of antibiotic production during microbial fermentation processes.

### 2.4. In Situ Localization of AmB Production

**Figure 4:**
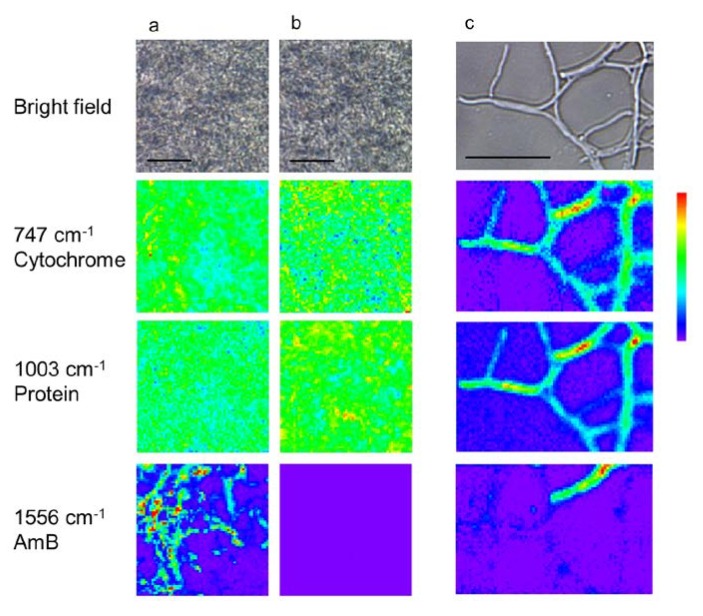
Raman images of mycelia and individual hyphae of *S. nodosus*. The images were obtained from the center of mycelia under amphotericin B (AmB)-inducing (**a**) and AmB-non-inducing (**b**) conditions. Magnified Raman images were obtained from a hypha in the edge area of a mycelium cultured under AmB-inducing conditions (**c**). Scale bar = 10 μm.

To elucidate the *in situ* distribution of AmB in *S. nodosus*, Raman images of cytochrome groups (747 cm^−1^), protein (1003 cm^−1^), and AmB (1556 cm^−1^) were constructed (Figure 4). In the center of the mycelia grown in liquid media (Figure 4a,b), proteins and cytochromes were distributed throughout the entire cell area, as seen in the bright field images. The cellular morphology is indistinguishable in the center of mycelia, because hyphae were closely-packed. In contrast, AmB was present only under the AmB-inducing conditions and shows local distribution within the measured area. There is no correlation between the localization of AmB and other biomolecules. To identify AmB production in single cells, the hyphae that expanded from mycelia grown in solid media were also analyzed by Raman imaging (Figure 4c). In these images, although the distribution of protein and cytochrome corresponds to regions that appear to be individual cells, AmB was heterogeneously distributed in the hyphae. These results suggest that AmB production is enhanced locally in the hyphae of mycelium, while the other biomolecules required for cell growth and structure are maintained and distributed over the entire area of the hyphae.

In general, antibiotic biosynthesis correlates with morphological development, cell density and growth phase in *Streptomycetes* [34,35]. Based on this phenomenon, we considered that the cells in the cell crowding center area accumulate much more AmB than those in edge area due to quorum sensing effect. In *S. nodosus*, the biosynthetic gene cluster for AmB (113 kbp) includes six large polyketide synthase genes, two cytochrome P450 enzyme genes, two ABC transporter genes, and genes involved in the biosynthesis and attachment of mycosamine. It was thought that the ABC transporters (AmphG and AmphH) formed a heterodimer that exported AmB from the cell for self-protection and resistance [8]. Nevertheless, the Raman images of mycelia and individual hyphae indicate that AmB is distributed inside mycelia and along hyphae, while they might contain both intracellular AmB precursors and accumulated AmB within the cells. Thus, we established that Raman imaging could provide intact and real-time localization of AmB production within cells. Since our Raman imaging technique allows us to obtain spatially resolved molecular information in living cells, this technique will be helpful to elucidate the mechanism of antibiotic biosynthesis associated with morphological development. Moreover, the ability to detect antibiotic synthesis *in situ* and at single-cell resolution could be useful for screening antibiotic-producing bacteria from environmental samples.

## 3. Experimental Section

### 3.1. Sample Preparation for Raman Microspectroscopy

In this study, *S. nodosus* (NBRC 12895) was used for Raman microspectroscopic analyses of AmB production. Yeast extract starch medium (0.2% yeast extract, 1% soluble starch) and 1/10 tryptic soy medium (0.3% Bacto Tryptic Soy Broth (Becton Dickinson, Franklin Lakes, NJ, USA)) were used as AmB production-inducing and -non-inducing liquid media, respectively. Spore suspensions were inoculated into 3 mL of each medium and then cultivated at 28 °C on a 180 rpm shaker for 5 days. Every 24 h after 2 days of cultivation, mycelia were recovered from the culture media and then washed with PBS to eliminate components from the medium that might obscure Raman signals. To image individual hyphae, streak culture from the glycerol stock was conducted on a 1/10 tryptic soy agar plate. After cultivation at 28 °C for 5 days, a coverslip was stamped on the colony and removed so that the hyphae were transferred to the coverslip. The mycelia were observed in wet conditions to keep them from drying out during measurement. The hyphae were examined by microscopy, and the individual hyphae were selected for Raman imaging based on the bright field image. As a standard of AmB, commercially available AmB powder (Sigma Aldrich, St. Louis, MO, USA) was dissolved in DMSO.

### 3.2. Raman Microspectroscopy and Imaging

All Raman spectroscopic measurements were carried out with a laboratory-built confocal Raman microspectrometer. A 532 nm line of an Nd:YAG laser (Compass 315M; Coherent Inc., Santa Clara, CA, USA) was used as the Raman excitation line. The laser beam was focused by a 100 × 1.4 NA objective lens (Plan Apo VC; Nikon Corporation, Tokyo, Japan) onto the sample placed on the stage of an inverted microscope (ECLIPSE Ti; Nikon Corporation, Tokyo, Japan). The back-scattered Raman light was collected by the same objective lens and measured with a spectrometer (MS3504i, 1200 lines/mm; SOL Instruments, Ltd., Minsk, Republic of Belarus) and a CCD detector (Newton DU920-M; Andor Technology Plc., Antrim, UK) according to previous report [24]. The lateral and depth spatial resolutions of the Raman imaging system were 0.3 and 2.6 μm, respectively. The laser power was set to 4–20 mW at the sample point. For the measurement of *S. nodosus* in suspension, 10 μL of cell samples were placed on a clean coverslip and sealed with nail polish. To image hyphae with high spatial resolution, the mycelia of *S. nodosus* were immobilized on a coverslip, as described in section 3.1, and the measurement field was determined by microscopic observation. To image mycelia and individual hyphae, the sample area was scanned at 0.5 and 0.3 μm pitch, respectively, using a piezoelectric stage (custom-made; Physik Instrumente GmbH & Co. KG, Karlsruhe, Germany). The exposure times were 1 s per point for the mycelia and 0.5 s per point for the individual hyphae.

### 3.3. Data Analysis

Raman spectra were acquired and processed by IGOR Pro software (WaveMetrics, Inc., Lake Oswego, OR, USA). After wavelength calibration using the Raman spectrum of indene, all spectra acquired by the Raman mapping experiment were processed by a singular value decomposition analysis for noise reduction, as described in previous reports [19,36]. After the data pre-processing, for measurement of area intensity of the Raman band, the fitted spectrum was processed by gauss fitting method in the spectral region containing the Raman markers associated with cytochrome, protein, and AmB (726–762 cm^−1^, 997–1009 cm^−1^, 1533–1568 cm^−1^, respectively). Their area intensities were calculated as peak area of each Raman band. Raman images were constructed as pseudocolor images from the area intensities of three Raman bands: 1003 cm^−1^ (protein), 747 cm^−1^ (cytochrome), and 1556 cm^−1^ (AmB).

### 3.4. Antifungal Activity Test

Culture medium (1 mL) was centrifuged at 2400× *g* for 5 min, and the supernatant was removed. Cell pellets were vortexed with ethanol to extract the bacterial components, including AmB. Paper discs (8 mm) were soaked with 70 μL of bacterial extract. The soaked discs were placed in Sabouraud Dextrose Agar plates seeded with 1-day broth culture of *Candida albicans* (NBRC 1594). The plates were incubated at 30 °C for 1 day. The antifungal activity was evaluated by the presence of an inhibition zone.

## 4. Conclusions

In summary, we were able to detect AmB in small volumes of *S. nodosus* cultures using Raman microspectroscopy *in situ*. Raman images reveal the heterogeneous distribution of AmB within the mycelia at single-cell resolution. Our results suggest that Raman microspectroscopy can provide information about intact antibiotic molecules that are produced by live cells, because the analysis does not require cell destruction. Additionally, Raman imaging can be useful to study the time-dependent change and cellular localization of antibiotic biosynthesis as well as the distribution and secretion of antibiotics from producer cells. Therefore, this technique could allow for routine assessments of microbial culture conditions and antibiotic production to facilitate more efficient antibiotic production. Moreover, Raman imaging could be employed to screen novel antibiotic candidates from marine samples. 
